# More research needed into long-term medical student mental health during COVID-19 pandemic and beyond

**DOI:** 10.1192/bjb.2021.49

**Published:** 2021-06

**Authors:** Anusha Mahesh Prabhu, Intishar Rashad

**Affiliations:** Imperial College London, UK. Email: anusha.prabhu@hotmail.com; Armed Forces Medical College, Dhaka Cantonment, Dhaka-1206, Bangladesh. Email: intisharrashad@gmail.com

The mental health of healthcare professionals has been significantly affected by the COVID-19 pandemic.^1^ We believe that more research focusing specifically on the long-term mental health of medical students is required. Medical students are an at-risk group, with a greater incidence of anxiety^2^ than the general population, and a higher prevalence of suicidal ideation than physicians and nurses.^3^ The risk is further compounded by being young,^1^ having higher awareness of disease severity, and possessing common personality traits such as maladaptive perfectionism and difficulty adapting to disruption in routine.^2^

Medical students often face a sense of duty to volunteer in hospitals in times of global health emergencies such as the current pandemic, which can bring particular emotional challenges. The subsequent higher risk of transmitting COVID-19 may cause increased social isolation. Uncertainty around medical education due to cancelled exams and placements also increases stress.^4^

Although a recent meta-analysis showed no difference in anxiety during COVID-19 in medical students,^2^ isolated studies worldwide have shown increased anxiety in those whose loved ones had contracted COVID-19 and who had interacted with COVID-19 patients.^4^

Higher levels of baseline stress and depression are negative predictors of poor mental health,^1^ so more research must be done to identify those especially at risk. Those with pre-existing mental health issues have reported decreased access to the usual support services during the pandemic.^1^ To our knowledge, there is no available literature exploring the impact of COVID-19 on medical students with pre-existing mental illness. Experiences in previous pandemics such as SARS show that long-term mental health issues such as alcohol misuse and post-traumatic stress symptoms can persist for several years in healthcare workers who have been quarantined or have worked in high-risk areas.^5^

The majority of current studies of mental health in COVID-19 have been cross-sectional, and few have focused on medical students. Longitudinal, large-scale, multi-country studies focusing on medical students of all age groups and investigating more variables, such as pre-existing mental illness, are required to identify those most at risk and the long-term effects on this population. The results of these studies could be used to improve future implementation of targeted medical student-specific mental healthcare interventions.^1^
